# Excess female adrenal androgen secretion is a consequence of *in utero* androgenic excess in sheep

**DOI:** 10.1530/RAF-26-0030

**Published:** 2026-06-08

**Authors:** S Ramaswamy, K Siemienowicz, A S McNeilly, W C Duncan, M T Rae

**Affiliations:** ^1^ School of Applied Sciences, Edinburgh Napier University, Edinburgh, UK; ^2^ Centre for Reproductive Health, Institute for Regeneration and Repair, Edinburgh BioQuarter, Edinburgh, UK

**Keywords:** adrenal, androgen excess, fetal development, polycystic ovary syndrome, polyendocrine metabolic ovarian syndrome

## Abstract

**Abstract:**

Excess androgenic steroid exposure during development in female sheep alters metabolic and reproductive function in postnatal life, with phenotypes reminiscent of health-relevant aspects of Polyendocrine Metabolic Ovarian Syndrome (PMOS) in women. We hypothesised that altered steroid exposure during *in utero* life would alter female postnatal adrenal function. Prenatal steroid excess was created by fetal steroid injections, on d62 and d82 of gestation, designed to separately create excess androgenic exposure (testosterone propionate, *n* = 14), oestrogenic exposure (diethylstilbestrol, *n* = 7) and glucocorticoid excess exposure (dexamethasone, *n* = 11) with controls (*n* = 11) treated with vehicle alone. Adrenal function tests and qPCR measurement of adrenal steroidogenic genes were the outcomes measured. In post-pubertal female offspring, specifically prenatal androgenic excess was associated with exaggerated testosterone secretory response to ACTH (*P* < 0.05), in the absence of alterations in cortisol secretion. Altered androgen secretory response was associated with increased *STAR*, *HSD3B1* and *HSD17B* mRNA (*P* < 0.05). Neither adrenal androgen secretion nor steroidogenic gene expression was altered prior to puberty. During fetal life (day 90 of gestation), only *STAR* was altered in prenatal androgenic excess females (*P* < 0.05), to a similar level as observed in a control cohort of male fetuses. We conclude that the postnatal, post-pubertal ovine adrenal gland is hyperandrogenic because of prenatal androgen excess.

**Lay summary:**

Steroid hormones are responsible for our development in fetal life. If there is too much or too little of a steroid hormone before we are born, it can affect our health in the future. If a female fetus is exposed to too much male-type hormone in the middle of pregnancy, after puberty, they develop features seen in women with Polyendocrine Metabolic Ovarian Syndrome (PMOS). The classic feature of PMOS is increased male-type hormones from the ovaries where cells in the ovaries are set up to make more male-type hormone. In women, half of all the male-type hormones come from the adrenal gland. We looked at the effects of giving more male-type hormones in the middle of pregnancy to female sheep fetuses as we know that they get the features of PMOS after puberty. In this study, we showed that the cells in the adrenal gland are also set up to make more male-type hormone after puberty. We checked if this was specifically due to the effects of male hormone exposure before birth and found that increased female hormone or stress hormone does not have the same effect. This means that it is likely not just the ovary whose function is altered in PMOS but also the adrenal gland.

## Introduction

Alterations in steroid exposure during development may lead to functional alterations in postnatal organ function, with potential health-relevant consequences. A well-studied paradigm of altered *in utero* steroid exposure/signalling is Polyendocrine Metabolic Ovarian Syndrome (PMOS) (formerly Polycystic Ovary Syndrome (PCOS); Teede *et al.* 2026). This syndrome encompasses a wide range of symptoms, which collectively ascribe it as the most common endocrinopathy in reproductive-aged women (Franks 1995). Insights into the origins of this condition have been made in a range of animal models, where reproductive and metabolic phenotypes reminiscent of human PMOS have been recreated by overexposure of female fetuses/pregnant dams to excess androgens (Abbott *et al.* 2005, Dumesic *et al.* 2007, Abbott *et al.* 2010, Padmanabhan *et al.* 2010, Roland *et al.* 2010, Hogg *et al.* 2012, Rae *et al.* 2013, Nicol *et al.* 2014). In this context, it is intriguing to note how cord blood of daughters of PMOS patients has been observed to have increased androgen concentrations (Barry *et al.* 2010), and increased anogenital distance in infants whose mothers have PMOS has been observed, suggesting involvement of increased androgenic exposure in humans possibly contributing to PMOS development (Barrett *et al.* 2018).

Whilst outcomes of studies examining adrenal androgen responses to ACTH in PMOS patients vary (Lachelin *et al.* 1979, Maas *et al.* 2016), there have been positive correlations observed between ovarian androgen production and adrenal androgen production, but not any differences in terms of cortisol response to ACTH associated with PMOS (Maas *et al.* 2016). Whilst supportive of the concept of dysregulated steroidogenesis at both ovarian and adrenal *loci*, this does not imply the same underlying mechanisms, and, given that androgens such as testosterone can be metabolised to other steroidal classes, effects observed in androgen excess pre-clinical animal models may mechanistically not be androgen mediated, but rather, via metabolism of excess applied androgenic steroids to steroids of different classes. This was highlighted in our earlier work demonstrating that pancreatic alterations resulting in hyperinsulinaemia were a direct effect of androgenic, but not oestrogenic excess, but androgen excess was nonetheless associated with increased oestrogen in the fetal circulation (Rae *et al.* 2013, Ramaswamy *et al.* 2016).

Utilising an ovine model of fetal steroid excess, we examined the functional, postnatal outcomes of dysregulated steroidal environments during development in terms of adrenal function. We hypothesised that prenatal androgen excess specifically would create a postnatal phenotype of hyperandrogenaemic adrenal function in response to trophic stimulation. We separately used two other steroid classes to compare responses to androgen excess since testosterone application also elevates fetal oestrogen concentrations (Rae *et al.* 2013), or treatments could conceivably cause elevated glucocorticoids via maternal or fetal stress. Our objectives were to determine the following: i) functional alterations in postnatal adrenal function because of prenatal androgen excess, ii) excess prenatal steroid specificity underpinning altered postnatal adrenal function and iii) early-life antecedents of postnatal adrenal function alterations to provide a mechanistic explanation.

## Materials and methods

### Ethics statement

Studies were approved by the UK Home Office and conducted under approved project licences PPL 60/4401, reviewed by the University of Edinburgh Animal Research Ethics Committee.

### Direct fetal steroid excess modelling

Mature Scottish Greyface ewes were fed to achieve a comparable body condition score (2.75–3) prior to oestrous cycle synchronisation. After a synchronised mating (Texel ram), animals were allocated at random to one of four experimental groups (testosterone propionate *n* = 14, diethylstilbestrol *n* = 7, dexamethasone *n* = 11 or vehicle control *n* = 11). Fetal steroid excess was achieved as previously described (Ramaswamy *et al.* 2016). Briefly, treatments were initiated on day 62 of gestation, post-sexual differentiation window. Maternal anaesthesia was induced by initial sedation using 10 mg xylazine i.m. (‘Rompun’, Baylor plc Animal Health Division, UK), followed by 2 mg/kg ketamine (i.v., Keteset, Fort Dodge Animal Health, UK), conducted under surgical aseptic conditions. Steroid signalling excess was driven by bolus application of diethylstilbestrol (DES;Connolly *et al.* 2013), dexamethasone (DEX 500 μg mL^−1^) or testosterone propionate (TP) (100 mg mL^−1^), dissolved in vegetable oil, and a 200 μL volume injected (20 G Quinke spinal needle, BD Biosciences, UK) via ultrasound guidance into the fetal flank on d62 of gestation. Control fetuses received 200 μL vegetable oil vehicle alone. This was repeated at d82 of gestation. Immediately after surgical procedure completion, all ewes were administered prophylactic antibiotics (Streptacare, Animalcare Ltd, UK, 1 mL/25 kg); no adverse effects of these procedures were observed in any experimental subjects. Animals were conventionally reared thereafter. No differences in birth weight associated with any treatment applied were observed (Ramaswamy *et al.* 2016), and at times of sampling, there were no differences in offspring weights.

### Tissue collection

Table 1 summarises all animal numbers in each group at each investigatory time point. Fetal tissue was collected on d90 of gestation as previously described (Rae *et al.* 2013, Ramaswamy *et al.* 2016). Adolescent female adrenal function tests were performed at 11 months postnatal age, and euthanasia/tissue collection was performed one week later. Adrenal function was also tested in a subset of these animals when they were pre-pubertal lambs (11 weeks postnatal age) from control and TP-treated groups; this subset was included in the 11-month adolescent cohort to permit pre- and post-pubertal comparisons. A further pre-pubertal subset of animals was euthanised at 12 weeks of age to provide tissue for molecular analyses. In all cases, euthanasia was achieved via barbiturate overdose; tissue samples were immediately snap-frozen and then stored at −80°C until downstream processing (Ramaswamy *et al.* 2016). These studies utilised offspring from both twin and singleton pregnancies (both twins injected with the same experimental treatment), but to avoid any possibility of genetic bias, we included only one animal from each pregnancy, selected at random, in analyses/adrenal function tests.

**Table 1:** Tissue collection timing and investigations conducted.

Treatment	11 months		11 weeks		90-day gestation	
	Investigation	*n*	Investigation	*n*	Investigation	*n*
Vehicle control	Adrenal function	11	Adrenal function	4	Adrenal mRNA	6
	Adrenal mRNA	6	Adrenal mRNA	5	Male fetal adrenals	6
Testosterone propionate	Adrenal function	14	Adrenal function	10	Adrenal mRNA	6
	Adrenal mRNA	6	Adrenal mRNA	7		
Diethylstilbestrol	Adrenal function	7				
Dexamethasone	Adrenal function	11				

### Adrenal function testing

A synthetic analogue of ACTH (Synacthen; tetracosactide acetate) was utilised to assess adrenal cortex function. A basal blood sample was withdrawn via a jugular vein indwelling catheter, and then immediately following this, a 2 mL volume of Synacthen (50 μg/mL), dissolved in sterile saline, was injected, followed by blood sample collection at 15 and 30 min post-Synacthen (Sid-Ahmed *et al.* 2013). Blood samples were immediately collected into heparinised tubes and then centrifuged at 1,000 ***g***, 4°C for 15 min, prior to collection of the plasma fraction and subsequent storage at −20°C until analysis. All animals were challenged and sampled at the same time on the same day in each case.

### Plasma analyte determinations

All plasma samples were co-extracted with the required standards and quality control samples before steroid determinations. For each, 200 μL of sample/standard were mixed vigorously for 10 min with 2 mL diethyl ether, and then, the mixtures were snap-frozen. The resultant organic fraction was decanted and evaporated to dryness under a stream of nitrogen at 37°C. Dried extracts were reconstituted in 250 μL assay buffer (PBS, 0.1% BSA, 1% Triton-X-100). Testosterone concentration was determined by in-house radioimmunoassay (Corker & Davidson 1978), using commercially available radioiodinated testosterone (MP Biomedicals, UK) and secondary antibody complex formation (SAPU, Carluke, Lanarkshire), as the separation method. Data were processed using AssayZap (Biosoft, UK), and intra- and inter-assay coefficient of variation was determined from quality control samples, <8% and <9%, respectively. Cortisol was measured by in-house ELISA, with intra- and inter-assay coefficient of variation of <7% and <10%, respectively.

### Determination of gene expression by (Q) RT-PCR

RNA was extracted, and Agilent Bioanalyzer was used for quality assurance (all samples RIN > 7.5). Post-reverse transcription (random nonamer primers, PrimerDesign ltd, UK), quantitative PCR was performed as previously described (Rae *et al.* 2013, Ramaswamy *et al.* 2016) utilising previously validated primer sets (Hogg *et al.* 2012), except for *HSD17B* (purchased pre-validated (PrimerDesign ltd) and *SRD5A2* (fwd: GCCGTTTCCAGTTGTATTCCT; rev: AGCAGGGTATTCAGCACAGTA); *MC2R* (fwd: ATGAAACACATTCTCCAATCTG; rev: AACGTTTTCCAAAATCTTGTAC) and *NR3C1* (fwd: AAGTCATTGAACCCGAGGTG; rev: ATGCCATGAGGAACATCCAT), designed in-house, commercially synthesised (Eurofins ltd, Germany) and validated prior to use. geNorm analysis identified a stable set of four housekeeping genes, *GAPDH, ATPSynth, RPS2* and *YWHAZ* (PrimerDesign ltd); thus, the geometric mean of these genes, Ct, was used as the normalisation reference. Negative controls consisted of RT-negative and template-negative reaction set; positive controls consisted of a pooled ovine adrenal cDNA sample.

### Statistical analysis

Analyses were conducted using GraphPad Prism, version 10.4.1 (GraphPad Software Inc., USA), accepting *P* values of <0.05 as statistically significant. A two-tailed, unpaired student *t*-test was used for two-group analyses, with >2-group comparisons by one-way ANOVA (Tukey’s post hoc test). Paired testing was used in the case of pre- and post-pubertal comparisons in the same animals. Adrenal function data were analysed by the area under curve (trapezoidal method).

## Results

### Adolescent offspring from fetal steroid excess studies

**Figure 1:**
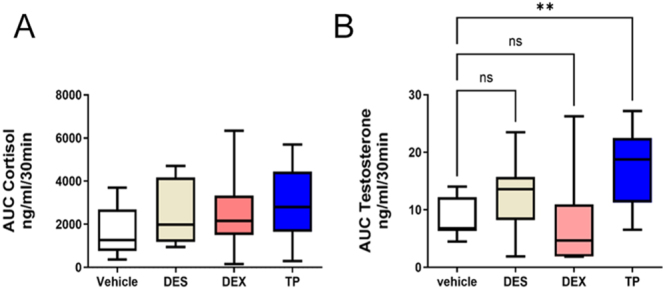
Increased testosterone in response to adrenal stimulation is specific to androgenic overexposure *in utero*. Intravenous ACTH (Synacthen) challenge was performed on 11-month-old female offspring from pregnancies where the fetus was directly exposed to increased androgen (TP, *n* = 14), oestrogen (DES, *n* = 7), glucocorticoid (DEX, *n* = 11) or vehicle (*n* = 11). Cortisol secretion did not differ between *in utero* treatment groups (A). Adrenal testosterone secretion was significantly elevated as compared to vehicle treatment only in the animals directly exposed to increased androgen during *in utero* development; steroids of other classes did not have this effect (B) (**P < 0.01).

**Figure 2:**
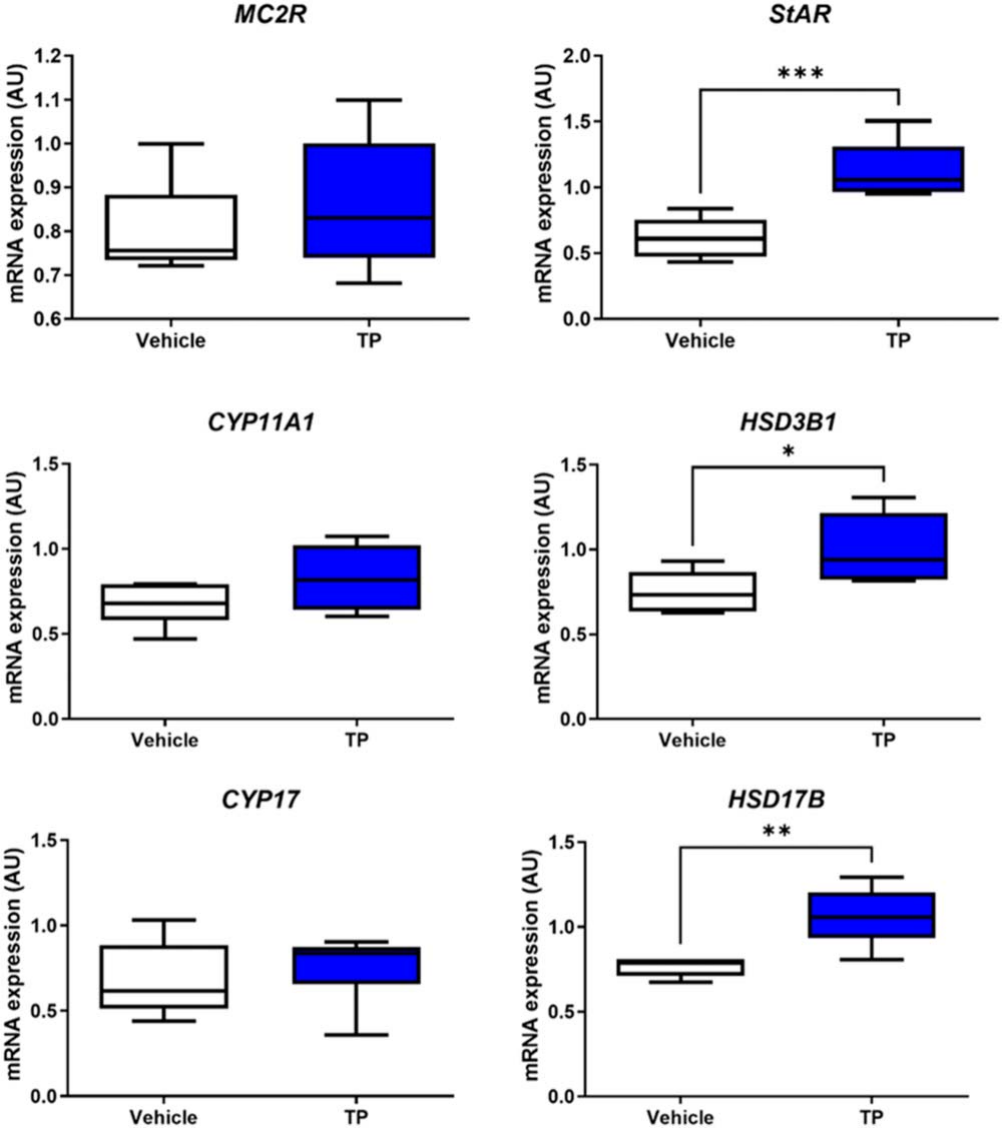
Basal adrenal steroidogenic-related gene expression during adolescence is altered by direct fetal androgen excess. qPCR was used to assess gene expression of steroidogenic-related genes in the adrenal cortex of 11-month-old female offspring from direct-fetal androgen excess pregnancies (TP, *n* = 6) and vehicle controls (*n* = 6). Fetal TP treatment was associated with elevated accumulation of mRNA’s encoding *STAR, HSD3B1* and *HSD17B* as compared to vehicle treatments (*P <0.05;**P < 0.01; ***P < 0.001).

To determine if post-pubertal adrenal function was altered by increased androgenic, oestrogenic or glucocorticoid signalling during development, a series of fetuses were treated with TP, DES or DEX during development, and at 11 months of age, female offspring were challenged with Synacthen (Fig. 1, panels A and B). No differences were observed between groups regarding cortisol secretion (Fig. 1, panel A). However, TP administration during fetal life was associated with exaggerated adolescent (11 months) secretion of testosterone over the 30 min post-Synacthen assessment period (Fig. 2, panel B); neither DES nor DEX exposure during development affected testosterone secretion in offspring. Observation of altered adolescent adrenal function only in prenatal androgen excess directed all downstream focus upon androgen excess animals.

qPCR was used to examine basal steroidogenic gene expression in the adrenal glands of these (fetal) TP-treated offspring and matched vehicle controls. We observed significantly greater expression of *STAR* (*P* < 0.001 as compared to vehicle), *HSD3B1* (*P* < 0.05 as compared to vehicle) and *HSD17B* (*P* < 0.01 as compared to vehicle) (Fig. 2).

### Pre-pubertal offspring from fetal steroid excess studies

**Figure 3:**
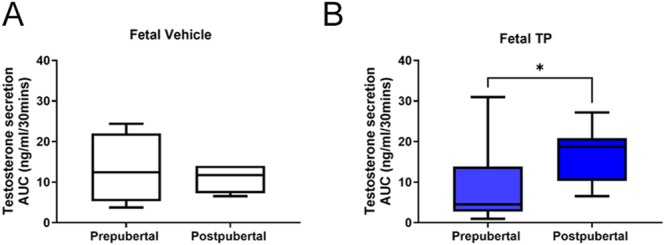
Exaggerated testosterone secretion as a legacy of prenatal androgen excess remains silent until after puberty in female offspring. A subset of female offspring from vehicle (*n* = 4) and TP pregnancies (*n* = 10) were tested for adrenal function before (11 weeks postnatal age) and after (11 months postnatal age) puberty. In vehicle-treated offspring, pubertal status did not affect adrenal testosterone secretion in response to ACTH challenge (A). In fetal TP-treated animals, post-pubertal adrenal challenge was associated with a significantly greater secretion of testosterone that was not evident in these same animals prior to puberty (B) (*P < 0.05).

**Figure 4:**
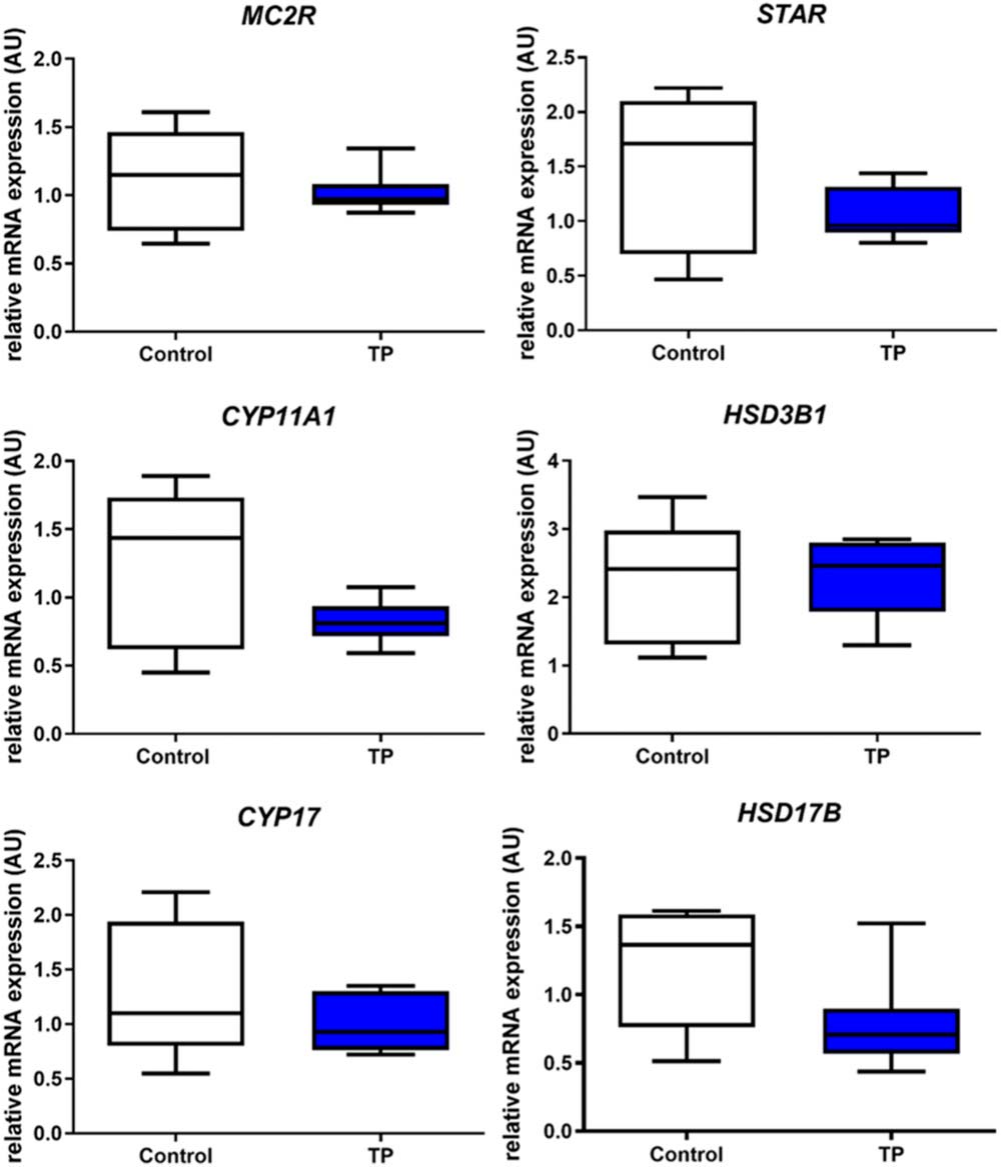
Pre-pubertal basal gene expression relevant to androgen synthesis is unaltered in pre-pubertal life by *in utero* androgen excess. At 3 months of age, unlike post-pubertal life, but in concert with lack of alteration to androgen secretion, we found no evidence of basal steroidogenic enzyme/trophic sensitivity relevant gene expression being affected by prenatal androgen excess (*n* = 5 control, 7 TP).

In a subset of animals, we performed identical adrenal testing at 11 weeks (pre-pubertal) age. We noted no effect of puberty observable in terms of ACTH-stimulated adrenal androgen secretion in vehicle offspring (Fig. 3, panel A). In offspring from fetal-TP treatments, post-pubertal adrenal androgen secretion (11-month-old) was significantly increased as compared to pre-pubertal (11-week-old) (*P* < 0.05) (Fig. 3, panel B). In tandem, there was no pre-pubertal alteration of the three key androgen synthetic genes (Fig. 4) that we observed to be increased at 11 months of age. It appears that the legacy of excess androgenic signalling during development as regards adrenal androgen secretion remained silent until after puberty.

### Examination of potential masculinisation of female fetal adrenal cortex by prenatal androgen excess

**Figure 5:**
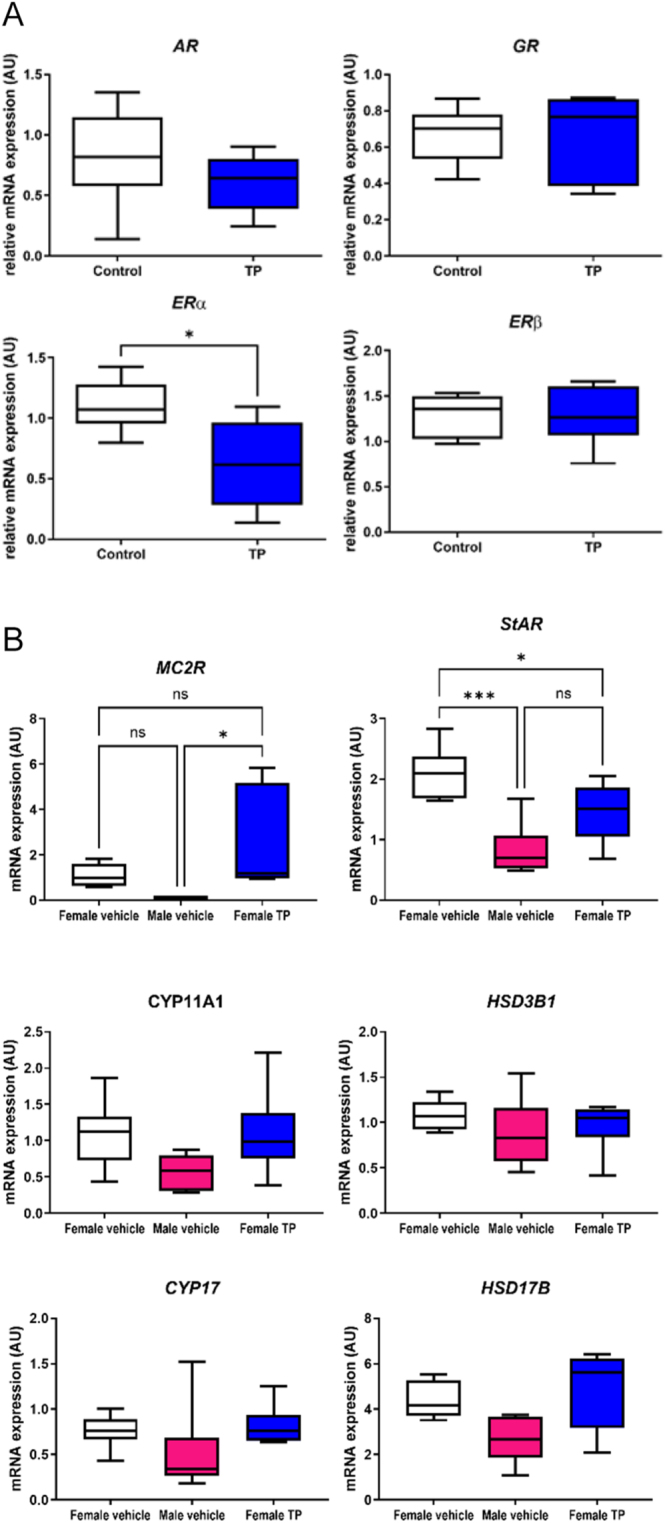
Female fetal androgen excess does not ‘masculinise’ the development of the adrenal cortex. Fetal adrenals were collected at d90 of gestation from vehicle-treated male (*n* = 6) and female animals (*n* = 6) and from TP-treated female animals (*n* = 6). mRNA accumulation of steroidogenic genes was measured by qPCR. (A) Steroid receptor expression was evident at the time of collection/treatment, and TP treatment was associated with reduced mRNA expression of ER*α*. (B) Male fetal adrenals displayed lower amounts of mRNA encoding *MC2R* and *STAR*, and significantly higher expression of mRNA encoding *NR3C1* and *SRD5A2*. Fetal female TP excess reduced *STAR* mRNA accumulation as compared to vehicle; no other effects were observed. Differing superscripts indicate significant difference (*P < 0.05; ***P < 0.001).

We considered that there may have been a masculinisation effect in the developing female adrenal gland due to prenatal androgen excess treatment, which remained silent until after puberty. Hence, we compared female fetal adrenal glands from vehicle treatments to male adrenals (also vehicle treated for comparison; *n* = 6) to determine sex differences, and in turn compared these to fetal adrenals from our *in utero* TP treatments, all at d90 of gestation (Fig. 5). Gene expression of androgen, glucocorticoid and oestrogen (α and β) receptors in the female fetal adrenal established potential for the fetal adrenal cortex to respond to the applied treatments – expression was unaffected by androgen excess, other than a significant depression of *ERα* (*ESR1*)expression (Fig. 5, panel A). Whilst we noted that male fetal adrenals displayed lower expression of mRNA encoding *MC2R* and *STAR*, and significantly higher expression of *NR3C1* and *SRD5A2* mRNA as compared to females, we found no apparent masculinisation of the female fetal adrenal. Only *STAR* was shifted in an apparently ‘male direction’ in response to excess androgen exposure in females. In TP-female fetal adrenals, *STAR* was reduced, as compared to vehicle-treated females (*P* < 0.05) (Fig. 5). However, this was not evident in pre-pubertal analyses (Fig. 4) and was in the opposing direction to the shift observed in post-pubertal analyses (Fig. 2).

## Discussion

In this study, adolescent, post-pubertal adrenal androgen secretion was increased because of excess androgen exposure in ovine female fetal life. This may have relevance for human conditions such as PMOS/adrenal androgen excess, where increased circulating concentrations of DHEAS and 11β-hydroxyandrostenedione are observed (Carmina *et al.* 1992, Maliqueo *et al.* 2009).

Zhou *et al.* (2005) demonstrated in a non-human primate model that excess prenatal androgen exposure was associated with adrenal hyperandrogenism (elevated DHEAS) in conjunction with increased corticosterone secretion. We observed that androgen overexposure in female sheep fetuses caused increased post-pubertal adrenal testosterone output in response to ACTH, in the absence of altered glucocorticoid secretion, indicative of species differences in responses to prenatal androgen excess. Previous work focusing upon the human HPA axis in PMOS has not identified alterations in terms of circulating concentrations of ACTH, but did observe, in agreement with our findings, that the adrenal response to ACTH, in terms of adrenal androgen secretion, was exaggerated (Azziz *et al.* 1998). No differences between PMOS and non-PMOS were observed in terms of cortisol response to ACTH, in further agreement with our results (Maas *et al.* 2016). Whilst in humans, the prevalent adrenal androgen is DHEAS, the adrenal cortex is also the origin of significant quantities of circulating testosterone (Yildiz & Azziz 2007), responsive to ACTH (Nakamura *et al.* 2009).

In sheep prenatal androgen excess models, maternal testosterone treatment elevated female fetal oestrogen concentrations in addition to testosterone concentrations (Veiga-Lopez *et al.* 2011, Rae *et al.* 2013); hence, to ascribe responses we specifically observed to androgenic excess during development, we used direct fetal injections of oestrogen agonist DES as a comparator treatment. We also included DEX treatment as a surrogate of stress-mediated glucocorticoid alterations and additionally as another adrenal steroid class, which could potentially be derived from the metabolism of applied androgens. Neither oestrogenic nor glucocorticoid excess during fetal life had observable effects upon postnatal adrenal androgen (or glucocorticoid) secretion. We conclude that the postnatal alterations in adrenal function are developmental androgen excess specific and, thus, focused the remainder of our studies upon androgenic excess.

To unravel mechanisms underpinning increased adrenal testosterone secretory response to ACTH, we examined the expression of key steroidogenic genes required for adrenal steroid synthesis in offspring from fetal-treated androgen excess pregnancies. At 11 months postnatal age, we observed significant elevations in mRNA encoding *STAR*, *HSD3B1* and *HSD17B*. The products of these genes are critical in terms of testosterone synthesis, indicative of the likelihood that, in agreement with circulating steroid measures in these animals, it was the androgen biosynthesis pathway that was specifically elevated in these animals. These mRNA-based observations were made in a basal, non-ACTH-challenged state and hence are indicative of an increased steroid synthetic capacity *per se* in the female offspring’s adrenal cortex exposed to excess testosterone during fetal life. In humans, it is considered likely that regulation of adrenal testosterone production is via *HSD17B* (Nakamura *et al.* 2009), underscoring the likelihood here that the observed increased *HSD17B* mRNA, in concert with elevated ACTH-stimulated testosterone production in the same animals, has functional relevance. In human PMOS, adrenal *CYP17* is elevated; however, a polymorphism in the *HSD17B5* gene may be involved in hyperandrogenism severity (Marioli *et al.* 2009), although this remains equivocal (Goodarzi *et al.* 2008). Since there was no alteration in mRNA expression in *MC2R* (encoding ACTH receptor), in tandem with no alteration in cortisol secretion, it appears unlikely that there was increased ACTH sensitivity, although since our experiments were not cortical zone specific, this cannot be excluded completely.

We then asked if the alterations of both adrenal function and gene expression were established in the developing fetal adrenal during androgen excess exposure, indicative of developmental permanency of effects in the adrenal. Reception of the steroid classes applied was evident in terms of mRNA expression of *AR*, *GR* and *ER* isoforms during fetal life. We observed that prenatal androgen excess was associated with depressed *ERα*, but as no effects of prenatal oestrogen excess signalling upon the endpoints measured here were observed, we cannot comment upon potential functional relevance of this observation. We noted that the female steroidogenic enzyme mRNA profile in the case of *STAR* predicted female adrenals to have a greater steroidogenic capacity *per se* than male adrenals during fetal life, in agreement with early studies of the sheep fetal adrenal (Ayromlooi & Essman 1978). Whilst there were clear fetal sex differences in levels of genes expressed, e.g. higher *MC2R* and *STAR* in females as compared to males, and elevated *NR3C1* and *SRD5A2* in males as compared to females, only in the case of *STAR* did exogenous testosterone treatment align treated females towards males. However, during fetal life, the effect of prenatal androgen excess was to depress *STAR* mRNA, in opposition to what we observe post-pubertally in such animals. In pre-pubertal animals from TP-fetal treatments, there were no basal alterations of steroidogenic gene expression relevant to androgen synthesis. When ACTH challenge was performed pre- and post-puberty, increased adrenal testosterone secretion was only evident post-puberty in fetal TP animals; vehicle control animals had similar testosterone secretion regardless of puberty status. Collectively, pre-pubertal and fetal data indicate that the observed gene expression and function alterations seen in post-pubertal life associated with prenatal androgen excess may not have a direct fetal adrenal antecedent. Regarding human parallels where prenatal androgen excess may occur, daughters of hyperandrogenaemic PMOS sufferers only begin to have increased basal and stimulated DHEAS concentrations during peri-puberty (as compared to non-PMOS daughters) (Maliqueo *et al.* 2009). We, of course, acknowledge that the direct translational relevance of our studies to such human conditions is limited by our measurement of testosterone as an androgenic output, not considered a clinically relevant human adrenal androgen.

What might then underpin the clearly altered functionality of adrenal glands exposed to androgen excess during development? We have previously observed that these same animals developed hyperinsulinaemia by 11 months postnatal age (Ramaswamy *et al.* 2016), thus coincident with adrenal hyperandrogenism. Insulin modulates adrenal androgen/adrenal precursor androgen synthesis/adrenal androgen sulphation (Hines *et al.* 2001) and, in PMOS, increases response to ACTH in terms of 17-hydroxy intermediate steroids (Tosi *et al.* 2011), whereas decreased adrenal androgens are observed during treatment of PMOS with resveratrol (Banaszewska *et al.* 2016) or troglitazone (Azziz *et al.* 2003), likely due to increased insulin sensitivity/decreased insulin. It is, therefore, an intriguing possibility that adrenal function alterations noted in the current study during adolescence may be a consequence of altered pancreatic function/hyperinsulinaemia (Rae *et al.* 2013, Ramaswamy *et al.* 2016), and we suggest this warrants further investigation.

In conclusion, we have demonstrated that a consequence of female prenatal androgen excess is post-pubertal adrenal hyperandrogenaemia, but that this may be a secondary effect, as opposed to primary *loci*, of altered *in utero* steroid exposure.

## Declaration of interest

The authors declare that there is no conflict of interest that could be perceived as prejudicing the impartiality of the work reported.

## Funding

This work was funded by a UK Medical Research Council (MRC) project grant (G0500717) to WCD and ASM and a MRC project grant (G0901807) to WCD, MTR and ASM. The funders had no role in study design, data collection and analysis, decision to publish, or preparation of the manuscript.

## Author contribution statement

Animal work was performed by ASM, MTR and WCD. Laboratory analyses were performed by SR, MTR, KS and WCD. SR, KS, ASM, WCD and MTR analysed datasets and prepared the manuscript.
